# Influence of Fenofibrate Treatment on Triacylglycerides, Diacylglycerides and Fatty Acids in Fructose Fed Rats

**DOI:** 10.1371/journal.pone.0106849

**Published:** 2014-09-08

**Authors:** Thomas Kopf, Hans-Ludwig Schaefer, Martin Troetzmueller, Harald Koefeler, Mark Broenstrup, Tatiana Konovalova, Gerd Schmitz

**Affiliations:** 1 Institute for Clinical Chemistry and Laboratory Medicine, University Hospital Regensburg, Regensburg, Germany; 2 Sanofi-Aventis Germany, R&D DIAB Div./Biomarker & Diagnostics, Frankfurt, Germany; 3 Core Facility Mass Spectrometry, ZMF, Medical University Graz, Graz, Austria; Steno Diabetes Center, Denmark

## Abstract

Fenofibrate (FF) lowers plasma triglycerides via PPARα activation. Here, we analyzed lipidomic changes upon FF treatment of fructose fed rats. Three groups with 6 animals each were defined as control, fructose-fed and fructose-fed/FF treated. Male Wistar Unilever Rats were subjected to 10% fructose-feeding for 20 days. On day 14, fenofibrate treatment (100 mg/kg p.o.) was initiated and maintained for 7 days. Lipid species in serum were analyzed using mass spectrometry (ESI-MS/MS; LC-FT-MS, GC-MS) on days 0, 14 and 20 in all three groups. In addition, lipid levels in liver and intestine were determined. Short-chain TAGs increased in serum and liver upon fructose-feeding, while almost all TAG-species decreased under FF treatment. Long-chain unsaturated DAG-levels (36:1, 36:2, 36:4, 38:3, 38:4, 38:5) increased upon FF treatment in rat liver and decreased in rat serum. FAs, especially short-chain FAs (12:0, 14:0, 16:0) increased during fructose-challenge. VLDL secretion increased upon fructose-feeding and together with FA-levels decreased to control levels during FF treatment. Fructose challenge of de novo fatty acid synthesis through fatty acid synthase (FAS) may enhance the release of FAs ≤16:0 chain length, a process reversed by FF-mediated PPARα-activation.

## Introduction

Fibrates such as fenofibrate (FF) are widely used in human medicine for their hypolipidemic effects [1], [2]. They belong to a larger group of molecules called peroxisomal proliferators (PPs) [3]. PPs activate the peroxisome proliferator activated receptor α (PPARα) and modulate genes involved in lipid metabolism [4]–[7]. PPARα activation decreases several mediators of vascular damage such as lipotoxicity, inflammation, ROS, endothelial dysfunction, angiogenesis and thrombosis through modulation of cell signaling related to microvascular dysfunction [2]. PPARα modulators are linked to species-specific regulation of genes important in cell growth and differentiation [8]. In rodents, PPARα activation is associated with peroxisome proliferation and hepatocellular carcinoma [9]. However, due to species differences at the molecular level of PPARα regulation, humans might be resistant to liver cancer induced by PPARα agonists [9]–[11]. Oxidative stress due to excessive H_2_O_2_-generation upon FF treatment leads to lipid peroxidation and oxidative DNA damage and hepato-carcinogenesis in rodents [12]–[15]. PPARα function is coupled to several co-activators that interfere with NFkB signaling pathways linked to carcinogenesis [16]. FF is found enriched in organs of absorption and elimination, the gut, liver and kidney [17]. The FIELD-Study (FF Intervention and Event Lowering in Diabetes) reported a robust and sustained decrease in plasma triglyceride-levels upon FF treatment [11]. In contrast, elevation of HDL-cholesterol and Apo A1 levels were less than expected and decreased progressively over the duration of the study[2]. A recent study suggests the possible application of HDL molecular composition for the stratification of patients that could potentially profit from FF treatment [18]. Recently it was shown that fish oil and FF treatment showed significant overlap in gene regulation, with fish oil down-regulating genes in cholesterol and fatty acid biosynthesis and FF down-regulating genes related to inflammation [19].

At the molecular level, PPARα forms a heterodimer with retinoid X receptor (RXR) upon agonist binding and stimulates the expression of various genes involved in FA β-oxidation and ω-oxidation, intracellular FA-transport and HDL-cholesterol metabolism [1], [20], [21]. Unsaturated FAs activate PPARα [22], and PPARα agonists inhibit the production of prostaglandin E2 (PGE_2_) in vitro [23]. Through FF activation PPARα decreases triglyceride (TAG) and hepatic VLDL-production by enhancing FA-oxidation in the liver. In addition, it facilitates TAG-removal by stimulating LPL-production and enhances hepatic lipoprotein uptake due to the suppression of apo-C-III production [1]. FF also increases LDL particle size and reduces the prevalence of small dense LDL (sd LDL) and non-HDL-cholesterol containing lipoproteins related to apoB. PPARα activation increases HDL-C by stimulating the production of apo A-I and apo A-II [24], [25].

Feeding previously fasted animals a low-fat/high-carbohydrate diet caused a marked induction of enzymes involved in catalyzing fatty acid desaturation [26], including ATP citrate lyase [27], FAS for lipogenesis [28], and SCD-1, which introduces a double bond in sn-9 position of a variety of fatty acyl CoA precursors [29]. SCD-1 is also a major player regulating the fatty acid composition of tissues [30], and SCD -/- mice are resistant to diet induced obesity [31]. Expression of SCD-1 in the liver of diabetic rats was found to be up-regulated, while Δ5-desaturase (FADS1) was not altered [32]. SCD-1 is regarded as a target for reversal of hepatic steatosis and insulin resistance [28]. FAS is a key enzyme of de novo lipogenesis, which catalyzes the terminal steps of FA-synthesis in the liver [28].

In rats, FF also induces carnitine-acetyltransferase (CAT), carnitine-palmitoyltransferase (CPT), fatty acyl oxidizing system (FAOS) and acetyl CoA oxidase 1 (Aco1) [12]. FF treatment of metabolic syndrome patients reduced plasma TAG-levels (30%) and also cholesterol (30%) in TAG-rich lipoproteins, together with a reduction of apo CIII and sdLDL and an increase in large LDL, but did not lower concentrations or turnover rates of NEFAs, nor did it change glucose or insulin responses to oral glucose challenge [33]. FF in this study modified FA-metabolism either in the liver or in TAG-rich lipoproteins but not in adipose tissue due to PPARα activation.

Dietary exposure to fructose may specifically provide lipid deposition in visceral adipose tissue, particularly in males, whereas glucose consumption appears to favor lipid deposition in subcutaneous adipose tissue [34]. Coingestion of fructose may elicit an unfavorable TAG-profile similar to fructose alone. Fructose feeding also induces ChREBP and increases the expression of lipogenic genes (FAS, ACC, SCD1).

The objective of this study was to investigate the effect of FF treatment on rats under metabolic overload conditions at the level of lipid species to get a more detailed insight into lipid metabolism than with the total level of lipid classes (like total TAG). Metabolic overload was induced in a rat model by fructose feeding, and serum, liver and jejunum samples of FF-treated animals and control groups were analyzed using ESI-MS/MS, ion trap LC-FT-MS and GC-MS techniques.

## Materials and Methods

### Study design

The animal studies were performed at Sanofi in Frankfurt-Höchst. The tissue and serum samples were taken from two different animal studies with the same basic design. The VLDL-data was derived from a third study. All experimental procedures were conducted according to the German Animal Protection Law. The animal studies were approved by the Sanofi-Aventis Deutschland GmbH institutional animal care and use committee and notified to the relevant authority. The institution is AAALAC accredited (AAALAC, 2012). Blood was drawn from the retro-orbital vein plexus under Isofluran CP (CP Pharma, Burgdorf, Germany) oxygen/nitric oxide anesthesia (3.5%, 2:1) Animals were anesthetized in a gas box. The animals were sacrificed by bleeding from the abdominal aorta under deep Isofluran CP (CP Pharma, Burgdorf, Germany) oxygen/nitric oxide anesthesia (3.5%, 2:1). The anesthesia was initiated in gas box and sustained with mask. In all cases treatment was applied between 7:30 and 8:30 a. m. and blood samples were drawn 1 hour after treatment.

#### Tissue samples (study 1)

Male Wistar rats (HsdCpb: WU) were obtained from Harlan Laboratories, NL and were used at the age of 10 weeks for the study. Animals were housed under controlled temperature (21–23°C), humidity (55%) in Macrolon type 4 cages (3 animals per cage) with a 12 h light-dark-circle with ad libitum access to drinking water. Animals were divided into 3 groups with group 1 receiving a standard diet (Ssniff), group 2 receiving a 10% fructose-challenge through drinking-water and group 3 receiving FF treatment through oral application and a 10%-fructose-challenge through drinking water. The study protocol ran for 14 days. In the FF treatment group, drug application (100 mg/kg p.o.) started at day 7 and lasted for 7 days. After the treatment period, the heart, the liver and the jejunum were extracted and stored at −20°C for lipid analysis.

#### Serum samples (study 2)

Male SPRD rats were obtained from Charles River Laboratories (Wilmington MA) and were used at the age of 10 weeks for the study. Animals were housed under controlled temperature (21–23°C), humidity (55%) in Macrolon type 4 cages (3 animals per cage) with a 12 h light-dark-circle with ad libitum access to drinking water. Animals were divided into 3 groups with group 1 receiving a standard diet, group 2 receiving a 10% fructose-challenge through drinking-water and group 3 receiving FF treatment through oral application and a 10%-fructose-challenge through drinking water. The study protocol ran for 20 days. In the FF treatment group, drug application (100 mg/kg p.o.) started at day 14 and lasted for 7 days. Serum samples were taken before start of the fructose treatment (day 0), after 14 days of fructose treatment but before FF treatment (day 14) and at the end of the FF treatment (day 20). Samples were stored at −80°C until lipid analysis.

#### VLDL-secretion (study 3)

Male Wistar rats (HsdCpb: WU) were obtained from Harlan Laboratories, NL and were used at the age of 10 weeks for the study. Animals were housed under controlled temperature (21–23°C), humidity (55%) in Macrolon type 4 cages (3 animals per cage) with a 12 h light-dark-circle with ad libitum access to drinking water. Animals were divided into 3 groups with group 1 receiving a standard diet, group 2 receiving a 10% fructose-challenge through drinking-water and group 3 receiving FF treatment through oral application and a 10%-fructose-challenge through drinking water. The study protocol ran for 14 days. In the FF treatment group, drug application (100 mg/kg p.o.) started at day 7 and lasted for 7 days. After 6 days the animals were fasted for approx. 23 h; then, Tyloxapol reagent (Sigma, 1:10 diluted with NaCl 0.9%) was applied and samples were taken after 1, 2, 4 and 6 hours.

### Clinical chemistry

Serum levels of cholesterol, triglycerides, and phospholipids, as well as the safety variables: aspartate transaminase (ASAT), alanine transaminase (ALAT), alkaline phosphatase (AP) were determined on a Roche Cobas 6000 at Sanofi in Frankfurt Höchst using the respective Roche clinical chemistry kits for human diagnostics. Assays were performed according to the instructions from the suppliers.

### Statistical analysis

Data are presented as mean±s.d.. Significant differences were calculated by an unpaired Mann-Whitney-U test. Two-point comparisons were performed. The two groups consisted of the fructose-free control group which was compared to the fructose-fed-group to establish the influence of the feeding itself. The second significance was determined between the fructose-fed-group and the Fenofibrate-treatment-group. For all statistical calculations, SPSS Statistics 19 software (IBM) was used. A P<0.05 was considered to be statistically significant (P<0.05 is denoted by *; P<0.01 is denoted by **).

### Lipid measurements

Analysis of TAG/DAG-species was performed according to published methods [35]. In brief, 30 µl serum were extracted with a mixture of methyl tert-butyl ether, methanol and water (MTBE/MeOH/H_2_O; 10:3:2.5, v/v/v; 9.77 ml total volume) according to Matyash et al. [36]. Lipid extracts were spiked with a 1 µM mix of 18 LIPID MAPS internal standards (LM 6000, LM 6001) and 5 µl of spiked samples were injected onto a Thermo 1.9 µm Hypersil GOLD C18, 100×1 mm HPLC column mounted in an Accela HPLC instrument (Thermo Scientific). The final concentrations of internal TAG- and DAG-standard were 1 and 3 µM respectively. Solvent A was water with 1% ammonium acetate and 0.1% formic acid, solvent B was acetonitrile/2-propanol 5:2 (v/v) with 1% ammonia acetate and 0.1% formic acid. The gradient ran from 35 to 70% B in 4 min, then to 100% B in another 16 min and held there for further 10 min. The flow rate was 250 µl/min. Data acquisition was performed by FT-ICR-MS (LTQ-FT, Thermo Scientific) full scans at a resolution of 200k and <2 ppm mass accuracy with external calibration. From the FT-ICR-MS preview scan, the four most abundant m/z values were picked in data dependent acquisition (DDA) mode, fragmented in the linear ion trap and ejected at nominal mass resolution. Normalized collision energy was set to 35%, repeat count was two and exclusion duration was 60 s. Quantitative analysis of data acquired by the platform described above was carried out by Lipid Data Analyzer [37].

Total hydrolysate FAs were analyzed as FAMEs by GC/MS according to a recently published method [38]. Briefly, lipids were extracted with chloroform/methanol according to Bligh and Dyer [39]. Derivatization was performed as follows. 10 µl serum or cell homogenate corresponding to 50 µg protein were methylated in PTFE screw capped Pyrex tubes. 1 µg each of C13:0 and C21:0 iso were added as internal standards in 50 µl methanol. 200 µl of acetyl-chloride were added and the sample was shaken vigorously at 20°C for 14 h. Afterwards 5 ml 6% potassium carbonate solution was added. FAMEs were extracted with 500 µl n-hexane. 100 µl of the n-hexane top layer was transferred into a 500 µl auto-sampler vial and crimped. Analysis of the NEFA fraction was performed after Dole-extraction [40]. The NEFA-fraction was separated by SPE-fractionation by a newly developed method [41]. Derivatization and GC/MS-analysis of this fraction was performed with the method described above.

## Results

Male Wistar rats were subjected to fructose feeding and subsequent treatment with fenofibrate (FF). Lipidomic analysis was performed using MS-techniques (for details see materials and methods).

### General values

The clinical chemistry values for total triglycerides, total cholesterol, total phospholipids and body/liver weight as well as food/water consumption are shown in table 1. Also added are the values for the study safety parameters aspartate aminotransferase (ASAT), alanine aminotransferase (ALAT) and alkaline phosphatase (AP). Values are given as means with s.d. where appropriate. The initial point of time was taken before the start of treatment, meaning the fructose-fed and the FF groups were on a fructose diet for one week at that time. These values were determined in the first rat study, in which the liver and jejunum lipid levels were measured. The final point of time was after one week of treatment with FF. Cholesterol, triglycerides and phospholipids were increased by fructose-feeding, while FF treatment reduced these levels below control levels. Body weight of the animals was approximately equal for the three groups at both times, while liver weight was slightly increased through fructose-feeding and highly increased after FF treatment. Water consumption was lower for the fructose-fed animals, while food consumption was almost doubled upon fructose-feeding and nearly back to control level upon FF treatment.

**Table 1:** Clinical chemistry data, organ weight, food and water consumption.

	initial			final		
	fructose-free	fructose-fed	FF	fructose-free	fructose-fed	FF
Cholesterol [mmol/l]	2.18±0.39	2.63±0.40	2.45±0.49	2.25±0.50	2.40±0.41	1.38±0.32*
Triglycerides [mmol/l]	2.17±0.53	2.73±0.93	2.66±0.40	1.98±0.58	3.11±0.95**	1.41±0.36**
Phospholipids [mmol/l]	2.09±0.23	2.64±0.31	2.62±0.27	2.19±0.39	2.68±0.30**	1.71±0.18**
Body weight [g]	350±24.41	360±13.98	353±15.82	370±25.84	382±19.75	376±18.11
Liver weight [g liver/animal]	-	-	-	14.95±1.35	17.49±1.41*	24.03±2.88**
rel. water consumption/100 g BW [g/d/animal]	26.93	22.64	22.31	22.59	17.63	19.45
abs food consumption [g/d/animal]	37.82	61.39	77.35	36.03	69.80	46.88
ASAT [u/l]	145.17±36.59	120.17±26.88	115.17±22.89	111.00±32.60	78.50±6.72*	81.50±39.52
ALAT [u/l]	76.67±7.84	71.83±15.85	68.50±10.63	70.33±9.81	61.83±10.38	54.50±12.93
AP [u/l]	228.33±31.43	225.67±18.44	216.50±38.21	207.17±29.19	200.33±21.82	248.33±36.38*

Values determined in study 1. Values given are mean values of the 6 animals/group with standard deviation included. Significant changes are indicated using *: P<0.05; **: P<0.01; ***: P<0,001. ASAT: aspartate aminotransferase, ALAT: alanine aminotransferase, AP: alkaline phosphatase (AP).

### Triacylglycerols and diacylglycerols

**Figure 1:**
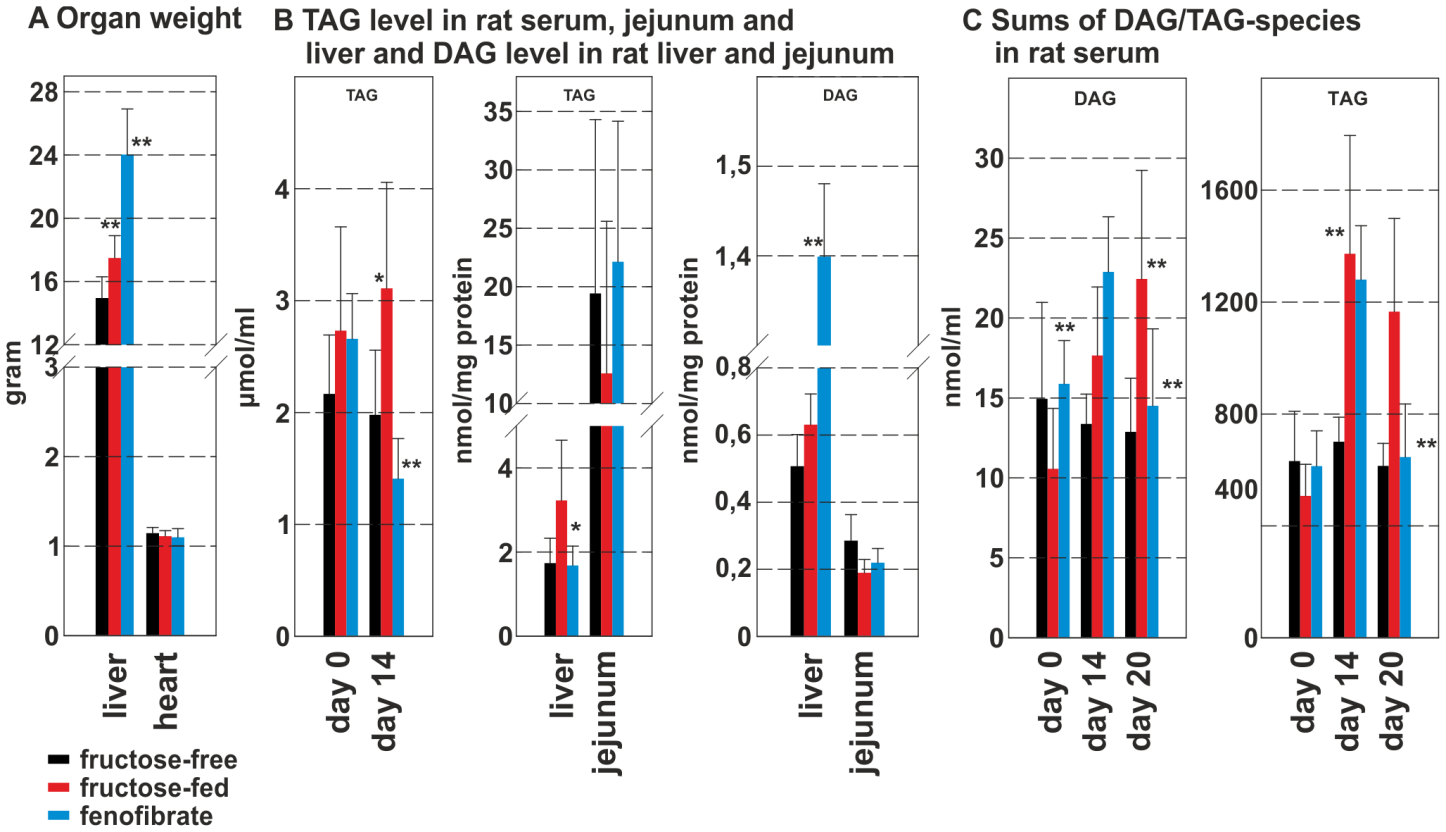
Tissue weights and overall TAG- and DAG-levels. **A** Organ weight of the animals after the end of treatment; **B** TAG-levels in rat liver, jejunum and serum and DAG-levels in rat liver and rat jejunum; **C** Sums of TAG- and DAG-species in rat serum. Tissue samples were taken during study 1, serum samples were taken during study 2; the control group is shown as black bar, the fructose-fed group is shown as red bar and the FF treated group is shown as a blue bar; values given are means±s.d.; significant changes are indicated using *: P<0.05; **: P<0.01; ***: P<0,001.

The weight of liver and heart of the three groups are shown in Fig 1A. The weight of the heart did not change upon fructose feeding or FF treatment, while the weight of the liver increased under fructose feeding and nearly doubled upon FF treatment. Absolute TAG-levels were determined in rat serum at the beginning (day 0) and at the end of the study (day 16, Fig 1B). A significant increase of TAGs under fructose-feeding, and a significant decrease by ∼25% under FF treatment was observed. The absolute levels of TAGs and DAGs (nmol/mg cell protein; Fig 1B) were analyzed in rat liver and jejunum at the end of the study. Without fructose-feeding rat jejunum contained about 10-fold higher TAG-levels than rat liver. Upon fructose-feeding TAG-levels decreased in the jejunum and increased in the liver, while FF slightly increased TAGs in the jejunum (∼20%) and decreased TAGs in the liver (∼50%) back to control group levels. DAG-levels in the liver increased upon fructose-feeding and more than doubled upon FF treatment. In contrast, baseline DAG-levels (Fig 1B) in the jejunum were 2-fold lower than in the liver, but did not change significantly under FF treatment. Fig 1C shows the sums of DAG- and TAG-species in the rat serum samples as an indicator for the total amount of DAGs/TAGs in rat serum. DAG-levels increased significantly by day 20 under fructose feeding and were reduced to control levels by FF treatment. The same was true for total TAGs, which increased upon fructose-feeding and decreased back to control levels by FF treatment.

### DAG-species analysis

**Figure 2:**
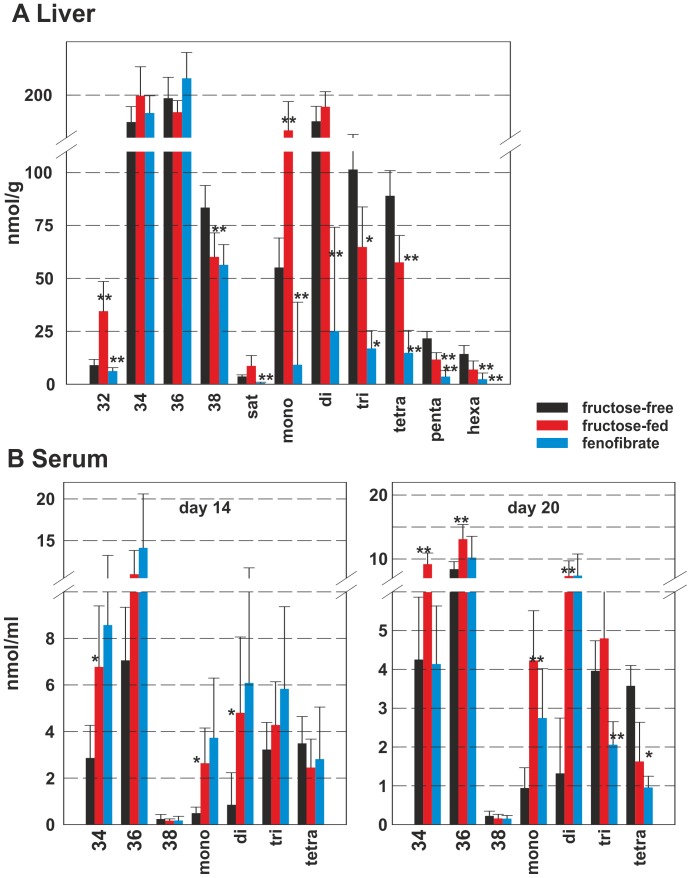
DAG-species in rat liver and serum. DAG-species in **A** rat liver and **B** rat serum. Values given are means±s.d. of the sums of the different chain lengths (C32 – C38) on the left and degree of desaturation (sat – hexa) on the right. Serum data is shown for day 14 and day 20; the control group is shown as black bar, the fructose-fed group is shown as red bar and the FF treated group is shown as a blue bar; significant changes are indicated using *: P<0.05; **: P<0.01; ***: P<0,001.

As a next step, individual DAG-species were determined in rat liver and rat serum at the three points of time. Fig 2A shows the DAG-species in rat liver. Given are the mean values of the sums of chain length of DAG-species (C32, C34, C36, C38) and the sums of degree of desaturation (saturated to hexa-desaturated). The complete data for the single DAG-species are shown in Fig. S1. During fructose-feeding, DAG-species containing 3 or more double bonds decreased, accompanied by an increase of species containing 2 or less double bonds. This increase is only present in the FA-species with 14, 16 or 18 carbons (C32, C34), while the C38-containing DAG-species decreased. FF-treatment caused variable changes of DAG-species. The short-chain DAG-species (32:0, 32:1 and 32:2; Fig. S1) decreased, as did DAG-species 34:0, 34:2 and 34:3, while DAG 34:1 increased. The longer-chain mono- and poly-unsaturated DAG-species 36:1, 36:2, 36:4, 38:3, 38:4 and 38:5 increased, while DAG 36:3, 38:2 and 38:6 decreased. The overall increase of DAG-levels was attributable to two species, DAG 34:1 and DAG 36:2, both may possibly contain FA 18:1.

It is interesting to note that only a few DAG-species (34:1, 34:2, 34:3, 36:2, 36:3, 36:4 and 38:4) were detectable in rat serum (Fig 2B). There was no difference between the groups on day 0. After 14 days of fructose feeding DAG 34:1 and 36:2 increased, which may also contain FA 18:1, while DAG 36:4 decreased. FF treatment decreased DAG-species 34:2, 34:3, 36:3 and 36:4 to below control levels. It is also notable that DAG 36:2 (18:1/18:1 or 18:0/18:2) is not influenced by FF treatment in rat serum.

### TAG-species analysis

**Figure 3:**
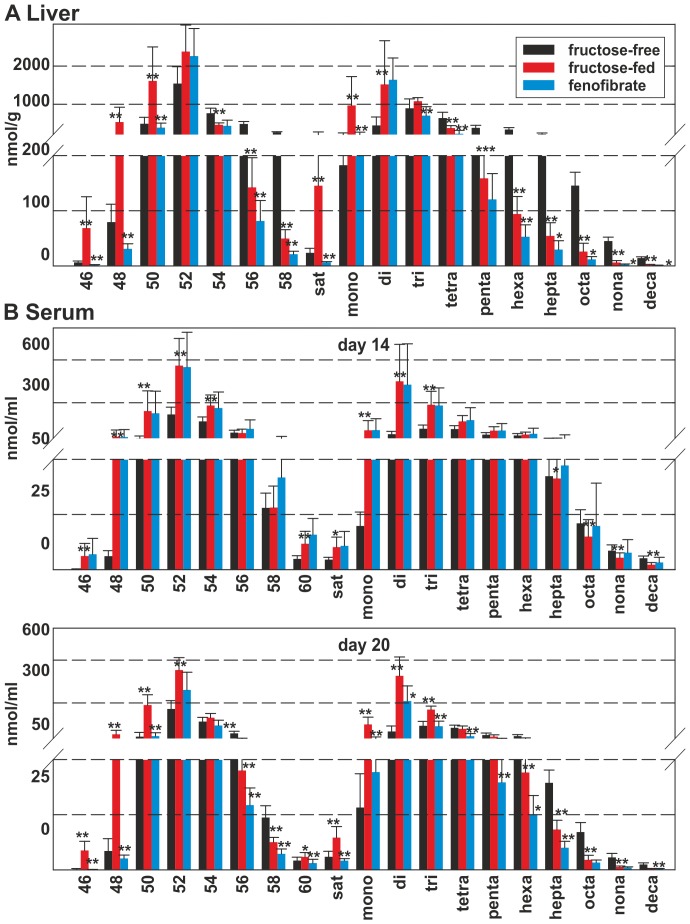
TAG-species in rat liver. Values given are means±s.d. of the sums of the different chain lengths (C46–C60) on the left and degree of desaturation (sat – deca) on the right. Serum data is shown for day 14 and day 20; the control group is shown as black bar, the fructose-fed group is shown as red bar and the FF treated group is shown as a blue bar; significant changes are indicated using *: P<0.05; **: P<0.01; ***: P<0,001.

TAG-species in the liver (Fig 3) are again displayed as means of sums of TAG-chain lengths (C46, C48, C50, C52, C54, C56, C58) and of sums of degree of desaturation (sat – deca). The levels of individual TAG-species are shown in Fig S2 (liver) and S3 (serum). Upon fructose-feeding TAG-species containing 4 or more double bonds decreased, while the species containing 3 or less double bonds increased. FF treatment decreased the concentration of almost all TAG-species. However, it is interesting to note that the absolute levels of TAGs in the FF-group were lower than at baseline, except TAG 50:2, TAG 52:1, TAG 52:2, TAG 54:2 and TAG 54:3.

In contrast to DAG-species, there were more TAG-species detectable in rat serum than in the liver (Fig S3). The groups showed only marginal differences in TAG-species levels before treatment. After 14 days of fructose most of the TAG-species increased in rat serum with the exception of the highly unsaturated long-chain TAG-species (56 and more carbons combined with 6 or more double bonds, Fig S3). FF treatment completely reversed this species shift. With the exception of TAG 52:2, all TAG-species were reduced to levels well below the levels in the rats of the untreated and unfed group. In total a clear trend is visible: The abundance of TAG-species C50, C52 and C54 is highest upon normal diet; fructose-feeding dramatically increased TAG-species C46, C48, C50 and C52 containing short-chain FAs (C12, C14, C16 and C18), while the TAG-species C56 and C58 containing long-chain FAs (C20 and C22) decreased.

### Total hydrolysate fatty acids species analysis

**Figure 4:**
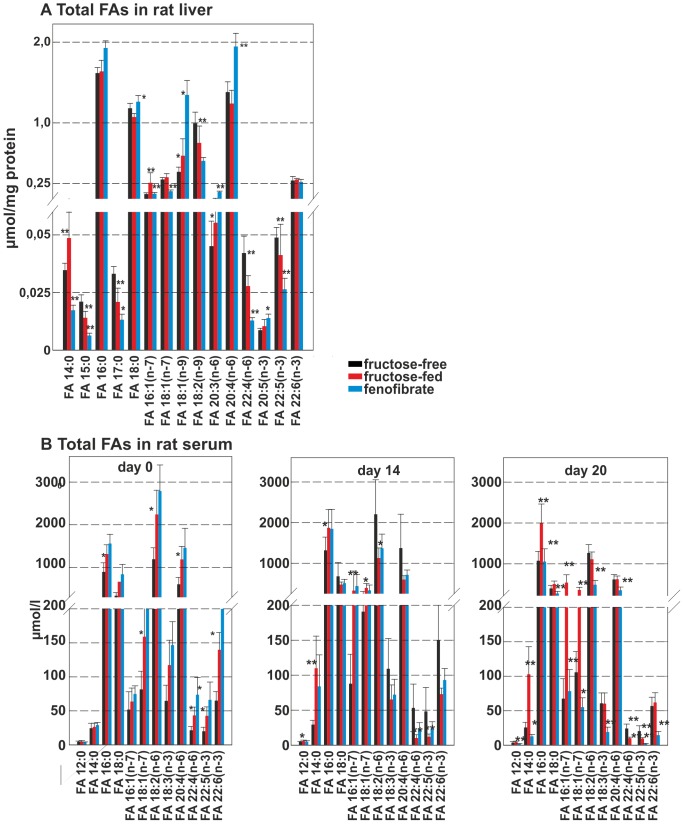
Total hydrolysate fatty acids in rat liver and serum. FA-species **A** total hydrolysate FA-species in rat liver **B** total hydrolysate FA-species in rat serum. The control group is shown as black bar the fructose-fed group is shown as red bar and the FF treated group is shown as a blue bar; values given are means±s.d.; significant changes are indicated using *: P<0.05; **: P<0.01; ***: P<0,001.

In the next set of experiments, total hydrolysate fatty acid species levels (FA) in rat liver (Fig 4A) were determined. Concentrations of liver total hydrolysate FAs during fructose-feeding were lower for FAs 15:0, 17:0, 18:2 (n-6), 22:4 (n-6) and 22:5 (n-3). Interestingly, FA 14:0, which is generally not released by fatty acid synthase increased by about 30% during fructose-feeding. Also increased were FAs 16:1 (n-7), 18:1 (n-9) and 20:3 (n-6), with the other species displaying no change. During FF treatment the FA-species 14:0, 15.0, 17:0, 16:1 (n-7), 18:1 (n-7), 22:4 (n-6) and 22:5 (n-3) decreased, while FAs 16:0, 18:0, 18:1 (n-9), 20:3 (n-6), 20:4 (n-6) and 20:5 (n-3) increased.

In the jejunum, total hydrolysate FAs generally decreased under fructose-feeding, FF treatment tended to increase FA 14:0, FA 18:0, FA 20:0, and FA 22:0, while the other total hydrolysate FA-species remained unchanged. None of these changes reached significance.

In addition, more NEFA-species were present in the jejunum, but no significant changes were seen upon fructose-feeding or FF treatment (data not shown).

The concentrations of total FAs in serum were determined in all three groups at day 0, day 14 (before FF treatment), and at day 20 (Fig 4B). Total FA-levels at day 0 were higher in the fructose-feeding group and the FF group. In the course of fructose-feeding, there was an increase in the short-chain FAs 14:0, 16:0, 16:1 (n-7) and 18:1 (n-7). In contrast, the abundance of long-chain FAs was reduced under fructose feeding, indicative of an impairment of elongase activity during metabolic (fructose) challenge. The increase of FA 18:1 (n-7) may be due to increased amounts of substrate FA 16:1 (n-7). Serum FA-levels of the fructose and the FF group were almost identical after 14 days of fructose-feeding and before FF treatment. The values at day 20 showed a further increase of short-chain FAs and a decrease of long-chain FAs upon fructose-feeding. FF treatment significantly reduced all FA levels, short-chain and long-chain, to or below baseline levels.

### Non-esterified fatty acid species analysis

**Figure 5:**
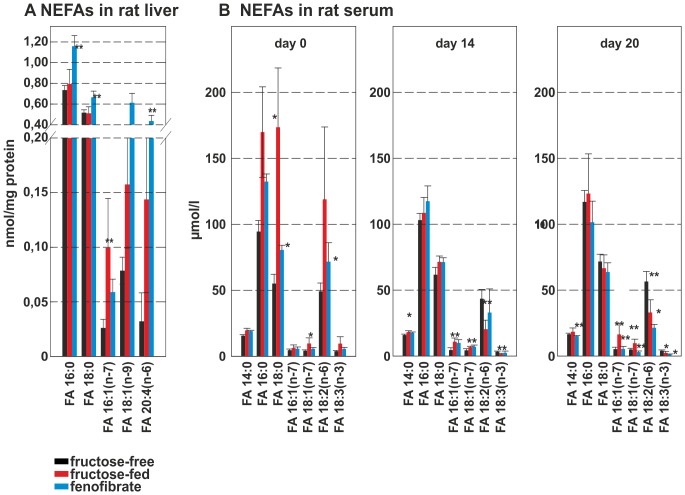
NEFA-species in rat liver and rat serum. **A** NEFA-species in rat liver homocysteine in rat serum; **B** NEFA-species in rat serum. The control group is shown as black bar, the fructose-fed group is shown as red bar and the FF treated group is shown as a blue bar; values given are means±s.d.; significant changes are indicated using *: P<0.05; **: P<0.01; ***: P<0,001.

The non-esterified fatty acid (NEFA)-species in the rat liver that significantly changed upon treatment are shown in Fig 5A. NEFAs increased during fructose-feeding, with significantly higher concentrations of FA 16:1 (n-7). Interestingly, during FF treatment FA 16:1 (n-7) decreased in rat liver, while FA 16:0, FA 18:0 FA, 18:1 (n-9) and FA 20:4 (n-6) increased significantly.

NEFAs at day 0 again showed higher levels for the fructose and the FF group (Fig 5B). During fructose feeding the NEFAs FA 14:0, FA 16:0, FA 16:1 (n-7), FA 18:0 and FA 18:1 (n-7) increased, while FA 18:2 (n-6) and FA 18:3 (n-7) decreased. NEFA-levels in the fructose-feeding and the FF group were identical before treatment. At day 20 the fructose-feeding group showed the same pattern compared to controls. Again, FF treatment reduced all NEFA levels to or below control group levels.

### Interrelation analysis of TAG-, DAG- and total FA-species

**Figure 6:**
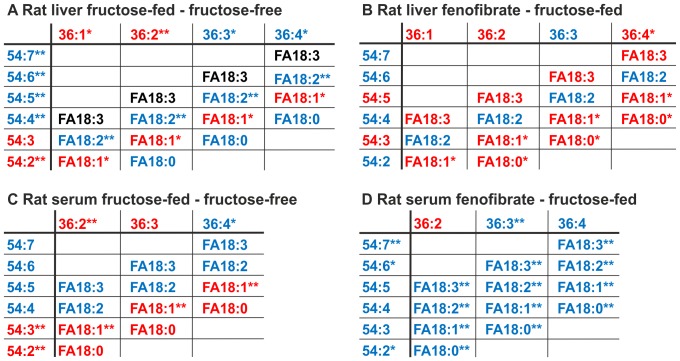
Alternate display format of TAG/DAG-species and FA-species. **A** fructose-feeding vs control in rat liver; **B** FF treatment vs Fructose-feeding in rat liver; **C** fructose-feeding vs control in rat serum; **D** FF treatment vs Fructose-feeding in rat serum; An algorithm was developed to generate the alternate display form of DAG/TAG-species. In the diagrams the species shown in red have increased levels between the two respective groups, while a blue color indicates a decreased level of the respective species between the two groups. A black coloring indicates no change of the species between the two groups. All species have been colored if the difference of levels between the two groups were >5% of the total amount of the higher level, regardless of the significance of the change. For clarity, only the sections of the diagram containing the combinations of DAG C36 With FA C18 to yield TAG C54 are shown. If there is a fatty acid-DAG combination present with the same direction of change as the corresponding TAG-species, then this combination seems at least to contribute to the composition of the TAG-species. Significant changes are indicated using *: P<0.05; **: P<0.01; ***: P<0,001.

The TAG/DAG- and FA-species were all quantified and the changes of species levels between the groups were plotted in an alternate display format (Fig 6). This figure contains just the section of TAGs with 54 C, DAGs with 36 C and FAs with 18 C to exemplify the principle. The complete displays are in Fig S4 and S5. With this information it is possible to determine graphically which FA and DAG-combinations might actually part of the corresponding TAG -species makeup. It is of course necessary to restrict the interpretation of this analysis to FA-species changes that reach significance.

We will give an example of this visualization. We have an increase of TAG 54:2 in the fructose-fed group compared to the controls in rat liver (Fig 6A). Considering the data of the DAG- and FA-species in this range (36 C for DAGs and 18 C for Fas), it seems most likely that TAG 54:2 consists of a combination of DAG 36:1 and FA 18:1 because both of those species are also increased, while FA 18:0, part of the potential combination DAG 36:2 and FA 18:0, is decreased. With these measurements of TAG/DAG-species it is possible to determine the most likely FA-combinations of the TAG/DAG-species (Fig S4).

It is also possible to determine the FA-species that converts a DAG-species into a corresponding TAG-species. For example, in the TAG-cluster containing 58 carbons (Fig S4), the species can be synthesized by esterification of DAG 38:5 with FA 20:0, 20:1, 20:2, 20:3, 20:4 or 20:5 respectively, to yield the resulting TAG-species (TAG 58:5, 58:6, 58:7, 58:8, 58:9 or 58:10). Of these six species TAG 58:9 and 58:10 decreased significantly under fructose feeding. The possible FA-DAG-combinations in which both components (FA and DAG) decreased are FA 20:4/DAG 38:5 for TAG 58:9; and FA 20:4/DAG 38:6 for TAG 58:10. These combinations are therefore likely to contribute to the compostion of those TAG-species. With this type of data analysis a relation of the up- or down-regulation of desaturation and elongation can be displayed graphically.

The same type of analysis has been performed for serum DAG/TAG/FA-species on day 20 (Fig S5). It is interesting to note that there were far less DAG-species present in rat serum but more TAG-species. Fructose-feeding induces the same pattern of increase/decrease of TAG-species in rat serum as in rat liver, as does FF treatment. Interestingly, all FA-species, except 20:3 decrease upon FF treatment.

**Figure 7:**
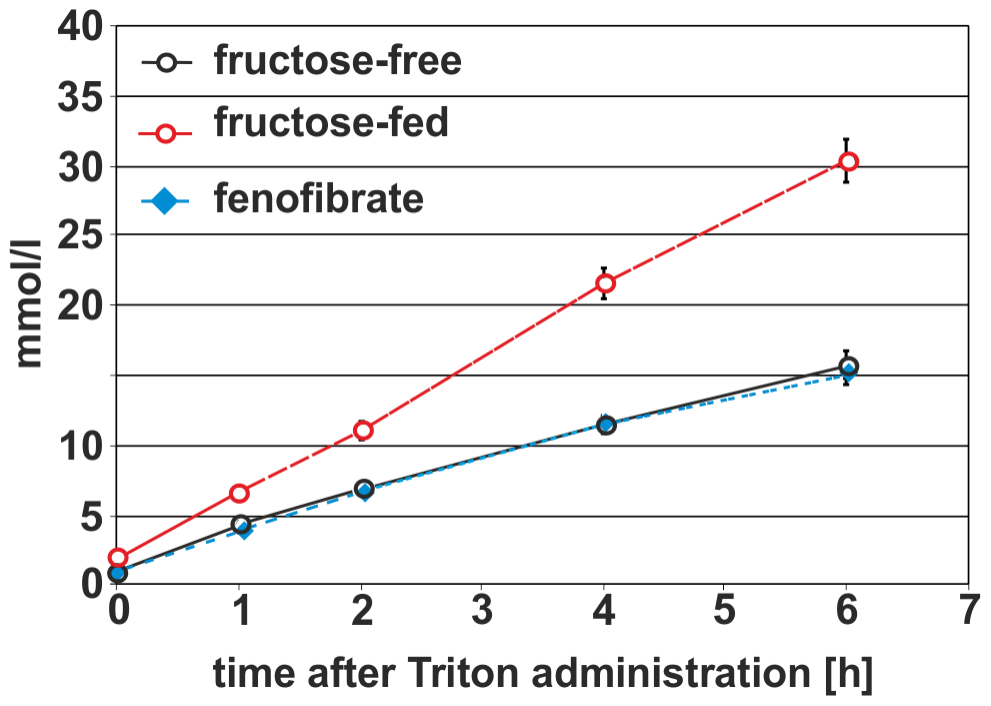
VLDL-secretion after FF treatment. The control group is shown in black, the fructose-fed group is shown in red and the FF treated group is shown in blue; values given are means±s.d.; significant changes are indicated using *: P<0.05; **: P<0.01; ***: P<0,001.

VLDL secretion was also monitored (Fig 7). VLDL secretion after Triton treatment was doubled upon fructose feeding, while FF treatment decreased VLDL-secretion back to control levels.

## Discussion

In the rat study presented here, most significant changes of lipid species occurred in rat liver, as a center of lipid synthesis and remodeling. TAGs are synthesized by esterification of DAGs through DGAT (consisting of two subtypes DGAT1 and DGAT2) with CoA-activated FAs being the substrates. The activity of DGAT is especially high in liver and intestine [42]. DGAT1 is most active at high substrate levels, which occur during high levels of lipolysis or during exposure to a high fat diet. DGAT2 on the other hand is most active at low substrate levels, which are connected to endogenous de novo FA-synthesis [43]. This endogenous synthesis is performed by fatty acid synthase, a multi-centered enzyme present in vivo as a dimer, which synthesizes fatty acids by incorporating C2-units until a chain-length of 16 carbons is reached and palmitate is released from the FAS-complex [44]. Fructose feeding increased FA 12:0 and 14:0, which are usually not liberated from FAS, in rat serum, while FF treatment reduced both species below control levels. Thus, fructose feeding seems to reduce the capacity of FAS for fatty acid binding of intermediates ≤C16:0 palmitate. This may be due to a metabolic overload of acetate caused by ingestion of fructose. The synthesis of other FA-species is then essentially achieved through elongation and desaturation of palmitate. Elongation involves elongases (ELOVL1/3/5/6) while desaturation is achieved by desaturases (SCD1-6, D5D, D6D) [45]. Under the influence of a diet high in carbohydrates (fructose-feeding) DGAT2 is the major enzyme active in the synthesis of TAGs as the storage form of FAs. In this study, TAG-levels in rat liver almost doubled upon fructose-feeding compared to standard diet and were decreased by FF-treatment to control levels. This indicates that DGAT2 inhibition is mediated by FF. It has been shown that SCD1 and DGAT2 are localized close to each other on the ER, indicating a participation of SCD1 in the synthesis of TAGs and the incorporation of MUFAs into TAGs [46]. This is in agreement with the presented data showing decreased FA16:1n7, the product of desaturation of FA16:0 by SCD1, in both NEFAs and total FAs. On the other hand, FA 18:1n9, which is also a product of SCD1, was increased in NEFAs and total FAs upon FF-treatment. This increase of FA 18:1n9 could be traced through most lipid species in liver and serum (e.g. CE 18:1) under FF-treatment. This might indicate that there is also SOAT1 activation by channeling oleate towards cholesteryl ester synthesis. Arachidonic acid (FA 20:4n6) is derived from linoleate through desaturation (D6D and D5D) and elongation (ELOVL5). In rat liver, FF-treatment significantly increased the level of the pro-inflammatory FA 20:4 (n-6) in total FAs and NEFAs, accompanied by a significant increase of the precursor FA20:3 (n-6) and a significant decrease of the corresponding precursor FA18:2 (n-6) in total FAs. In the NEFA-fraction, the FA18:2 (n-6) concentration remained unchanged compared to fructose-feeding, indicative of elongase (ELOVL5) and desaturase (D5D) activation towards the n-6-series induced by FF-treatment in rat liver. Most FA-species of the anti-inflammatory n-3-series decreased or remained below detection levels.

It is interesting to note that the weight of the liver increases significantly under FF treatment. The underlying mechanism is unclear. It might be due to the increased metabolic action induced by PPARα-activation resulting in an increased hepatic activity.

In order to verify the hypothesis described above, the linkage of transcriptional data to the lipid metabolite profiles is attempted as an extension of the present study.

## Summary

The present study presents a detailed investigation of lipid species during fructose challenge and fenofibrate treatment in rats. To our knowledge, it is the most complete lipidomic analysis on FF effects conducted to date. In the first part, we determined the levels of TAG- and DAG-species as well as the amounts of total hydrolysis FA- and NEFA-species in rat liver and rat serum. Short-chain TAGs increased in serum and liver upon fructose-feeding and almost all TAG-species decreased under FF treatment, while longer-chain, more unsaturated DAG-levels (36:1, 36:2, 36:4, 38:3, 38:4, 38:5) increased upon FF treatment in rat liver and decreased in rat serum. FAs, especially short-chain FAs (12:0, 14:0, 16:0) and VLDL secretion increased during fructose-challenge and decreased to control levels during FF treatment. Fructose challenge of de novo fatty acid synthesis through fatty acid synthase (FAS) may enhance the release of ≤16:0 chain length fatty acids, a process that is reversed by FF-mediated PPARα-activation.

In addition, we present a correlation diagram of the data that combines theoretical compositions of TAG and DAG with relative abundances of TAG, DAG and FA species, thereby providing a qualitative view on probable TAG and DAG compositions.

## Supporting Information

Figure S1 **DAG-species in rat liver and rat serum.** The control group is shown as black bar, the fructose-fed group is shown as red bar and the FF treated group is shown as a blue bar; values given are means±s.d.; significant changes are indicated using *: P<0.05; **: P<0.01; ***: P<0,001.
(TIF)

Figure S2 **TAG-species in rat liver.** The control group is shown as black bar, the fructose-fed group is shown as red bar and the FF treated group is shown as a blue bar; values given are means±s.d.; significant changes are indicated using *: P<0.05; **: P<0.01; ***: P<0,001.
(TIF)

Figure S3 **TAG-species in rat serum.** The control group is shown as black bar, the fructose-fed group is shown as red bar and the FF treated group is shown as a blue bar; values given are means±s.d.; significant changes are indicated using *: P<0.05; **: P<0.01; ***: P<0,001.
(TIF)

Figure S4 **Alternate display format of TAG/DAG-species and FA-species.** **A** fructose-feeding vs control in rat liver; **B** FF treatment vs Fructose-feeding in rat liver; An algorithm was developed to generate the alternate display form of DAG/TAG-species. In the diagrams the species shown in red were increased upon fructose-feeding compared to baseline, while a blue color indicates a decreased level of the respective species. For clarity, only the sections of the diagram containing the combinations of DAG C36 With FA C18 to yield TAG C54 are shown. If there is a fatty acid combination present with the same direction of change as the corresponding TAG-species, then this combination should be at least the biggest contributor to the TAG-species. Significant changes are indicated using *: P<0.05; **: P<0.01; ***: P<0,001.
(TIF)

Figure S5 **Alternate display format of TAG/DAG-species and FA-species.** **A** fructose-feeding vs control in rat serum; **B** FF treatment vs Fructose-feeding in rat serum; An algorithm was developed to generate the alternate display form of DAG/TAG-species. In the diagrams the species shown in red were increased upon fructose-feeding compared to baseline, while a blue color indicates a decreased level of the respective species. For clarity, only the sections of the diagram containing the combinations of DAG C36 With FA C18 to yield TAG C54 are shown. If there is a fatty acid combination present with the same direction of change as the corresponding TAG-species, then this combination should be at least the biggest contributor to the TAG-species. Significant changes are indicated using *: P<0.05; **: P<0.01; ***: P<0,001.
(TIF)
