# Supplementary figures and images for: Influence of Fenofibrate Treatment on Triacylglycerides, Diacylglycerides and Fatty Acids in Fructose Fed Rats
Source: PLoS One. 2014 Sep 8;9(9):e106849. doi: 10.1371/journal.pone.0106849 (PMC4157811; doi:10.1371/journal.pone.0106849)

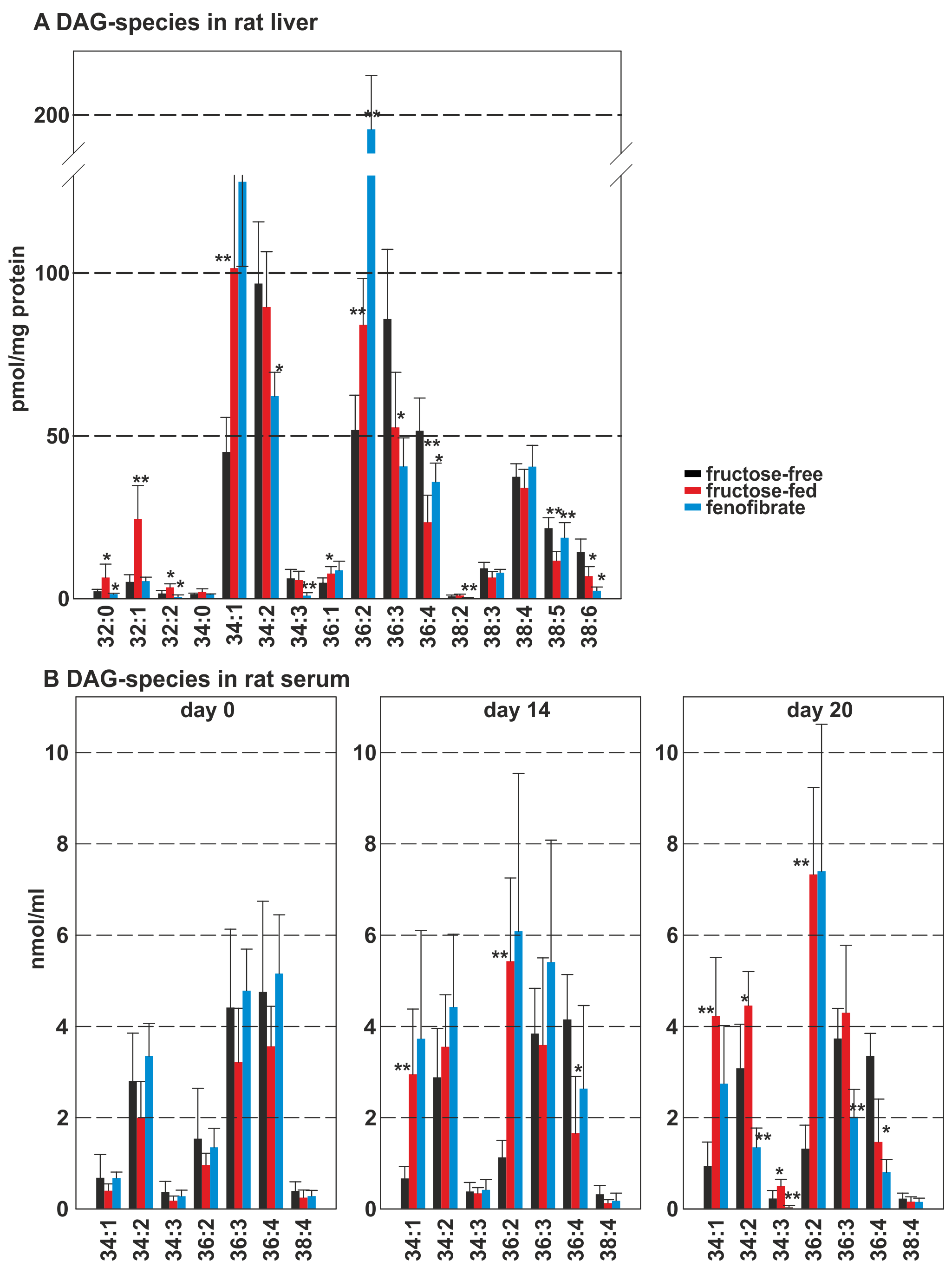

Supplement: Figure S1 — DAG-species in rat liver and rat serum. The control group is shown as black bar, the fructose-fed group is shown as red bar and the FF treated group is shown as a blue bar; values given are means±s.d.; significant changes are indicated using *: P<0.05; **: P<0.01; ***: P<0,001. (TIF) [file pone.0106849.s001.tif]

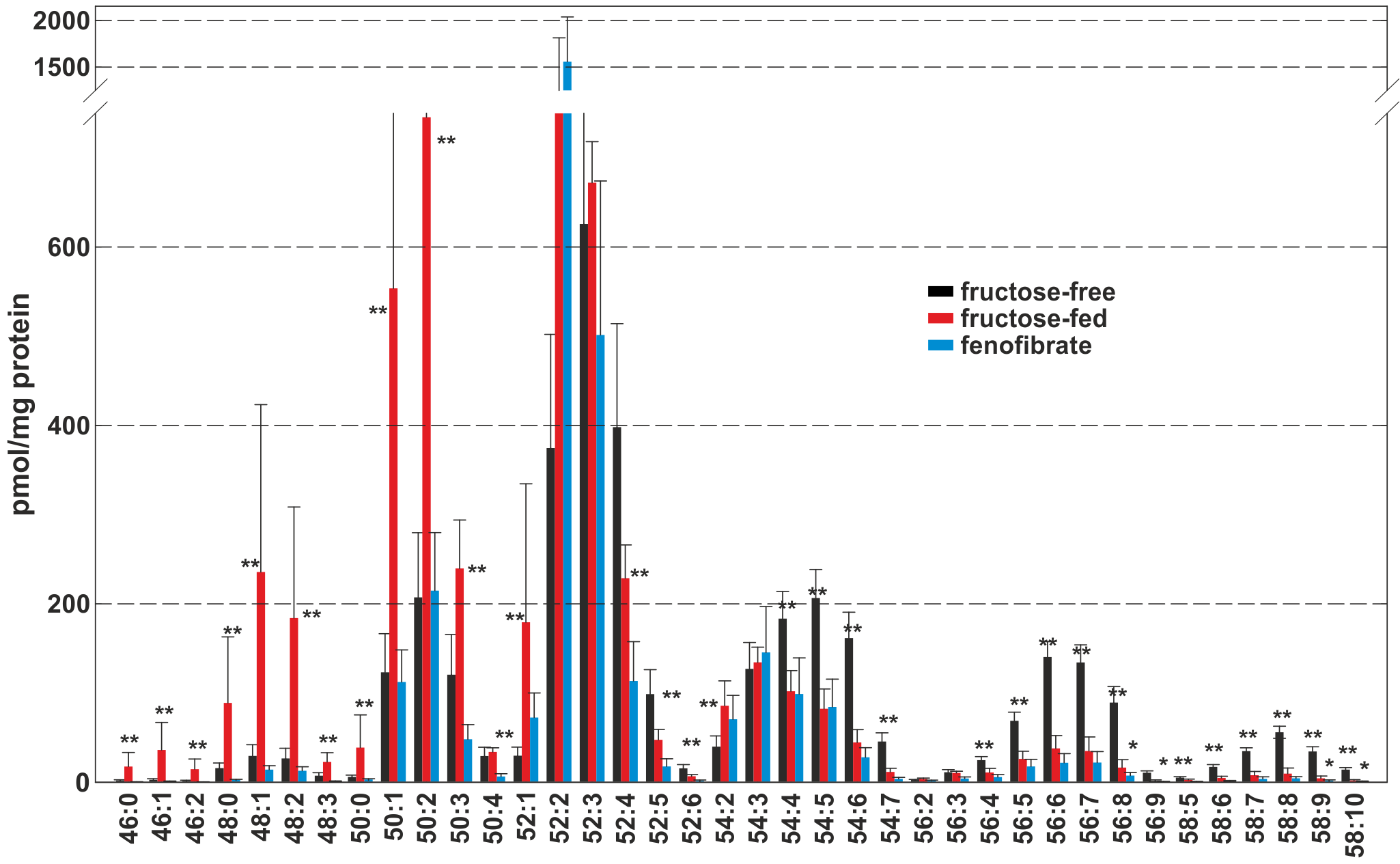

Supplement: Figure S2 — TAG-species in rat liver. The control group is shown as black bar, the fructose-fed group is shown as red bar and the FF treated group is shown as a blue bar; values given are means±s.d.; significant changes are indicated using *: P<0.05; **: P<0.01; ***: P<0,001. (TIF) [file pone.0106849.s002.tif]

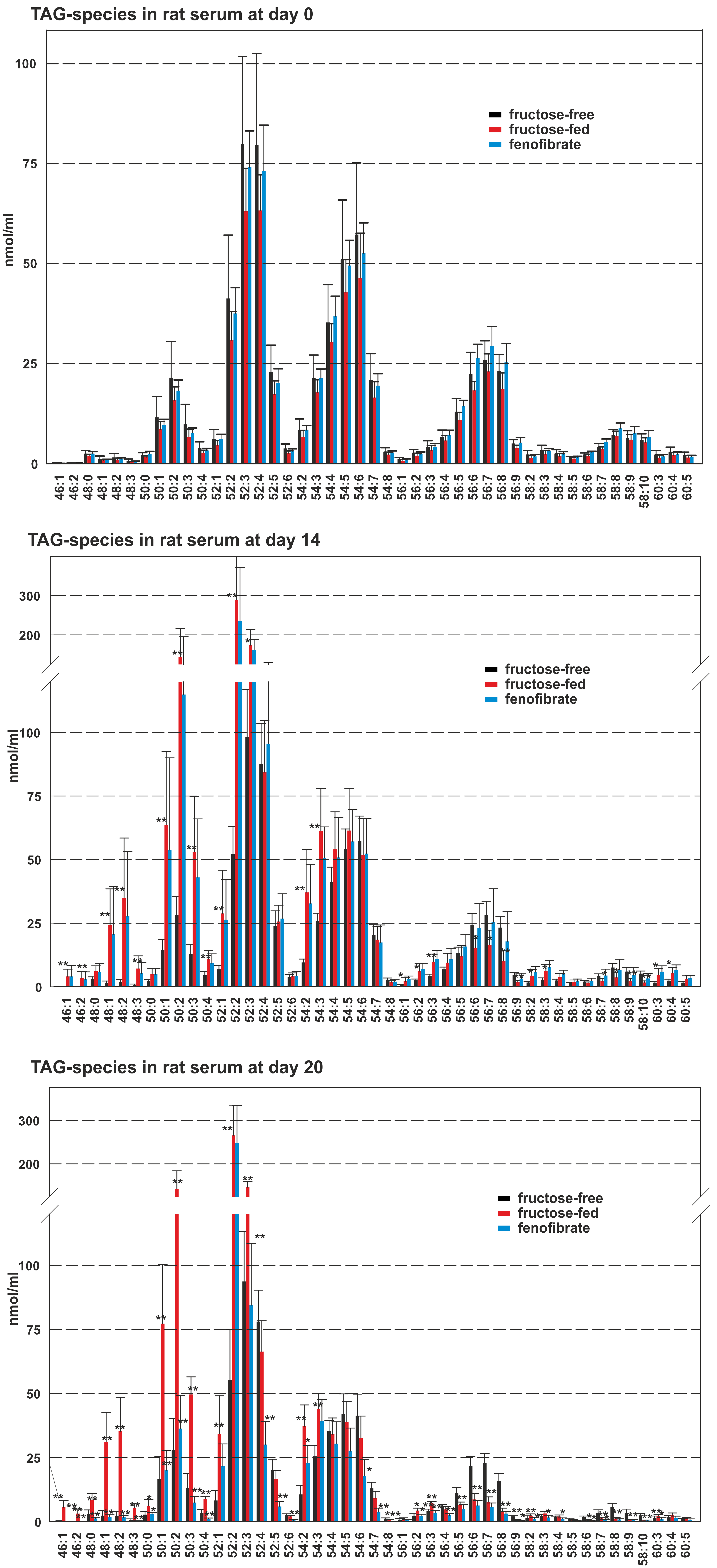

Supplement: Figure S3 — TAG-species in rat serum. The control group is shown as black bar, the fructose-fed group is shown as red bar and the FF treated group is shown as a blue bar; values given are means±s.d.; significant changes are indicated using *: P<0.05; **: P<0.01; ***: P<0,001. (TIF) [file pone.0106849.s003.tif]

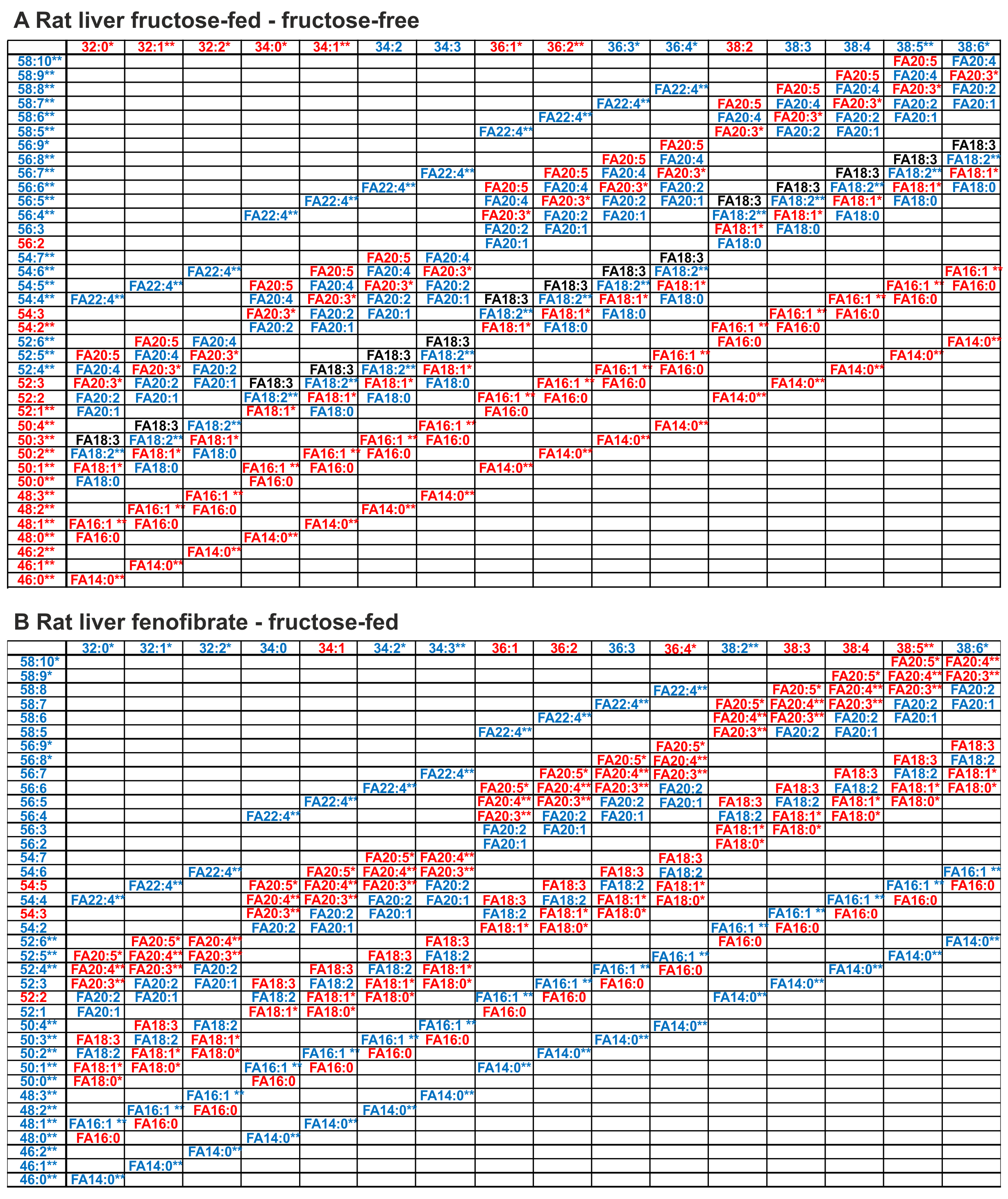

Supplement: Figure S4 — Alternate display format of TAG/DAG-species and FA-species. A fructose-feeding vs control in rat liver; B FF treatment vs Fructose-feeding in rat liver; An algorithm was developed to generate the alternate display form of DAG/TAG-species. In the diagrams the species shown in red were increased upon fructose-feeding compared to baseline, while a blue color indicates a decreased level of the respective species. For clarity, only the sections of the diagram containing the combinations of DAG C36 With FA C18 to yield TAG C54 are shown. If there is a fatty acid combination present with the same direction of change as the corresponding TAG-species, then this combination should be at least the biggest contributor to the TAG-species. Significant changes are indicated using *: P<0.05; **: P<0.01; ***: P<0,001. (TIF) [file pone.0106849.s004.tif]

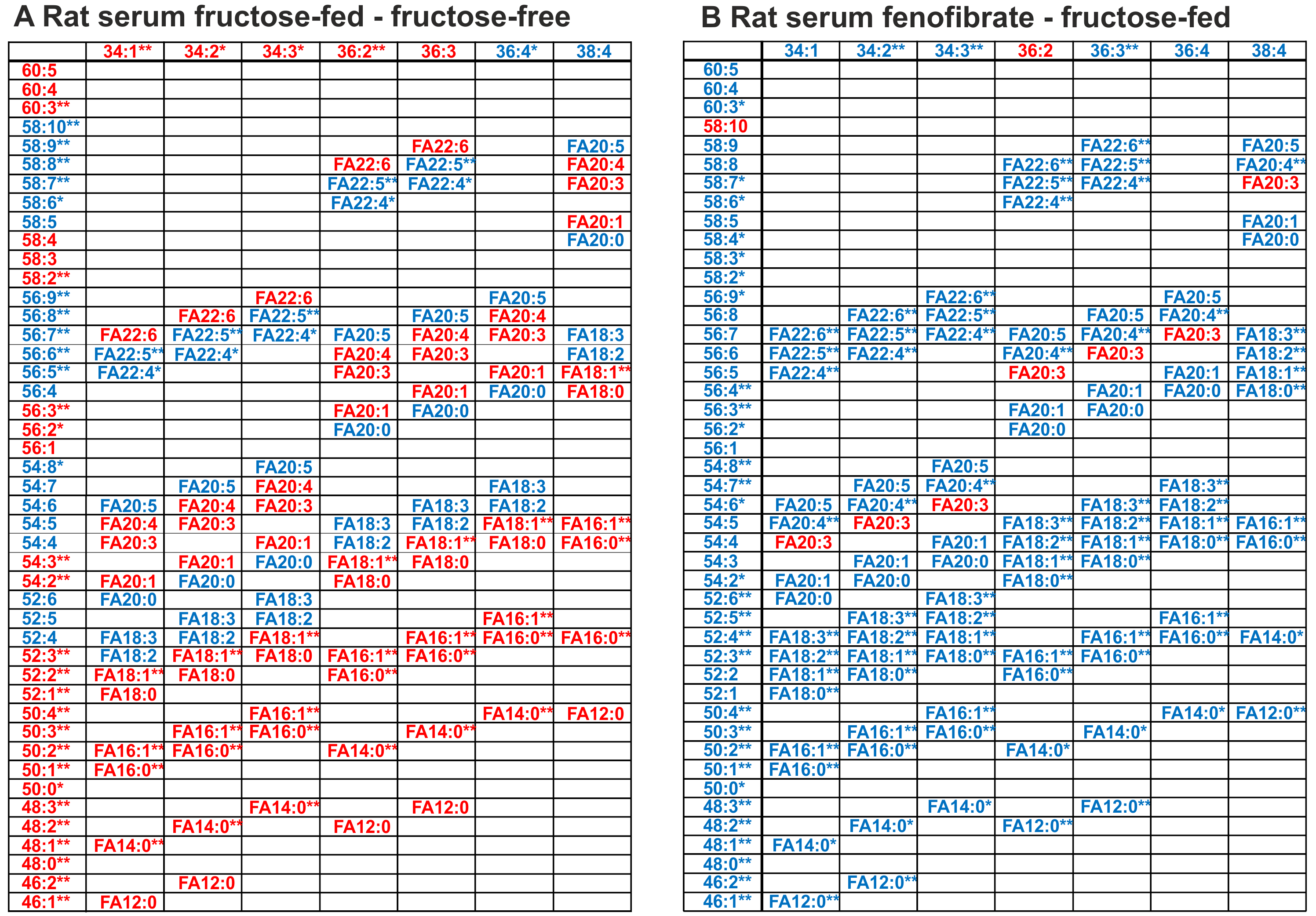

Supplement: Figure S5 — Alternate display format of TAG/DAG-species and FA-species. A fructose-feeding vs control in rat serum; B FF treatment vs Fructose-feeding in rat serum; An algorithm was developed to generate the alternate display form of DAG/TAG-species. In the diagrams the species shown in red were increased upon fructose-feeding compared to baseline, while a blue color indicates a decreased level of the respective species. For clarity, only the sections of the diagram containing the combinations of DAG C36 With FA C18 to yield TAG C54 are shown. If there is a fatty acid combination present with the same direction of change as the corresponding TAG-species, then this combination should be at least the biggest contributor to the TAG-species. Significant changes are indicated using *: P<0.05; **: P<0.01; ***: P<0,001. (TIF) [file pone.0106849.s005.tif]
